# Healthy people in healthy premises: the Finnish Indoor Air and Health Programme 2018–2028

**DOI:** 10.1186/s13601-020-0308-1

**Published:** 2020-01-17

**Authors:** Jussi Lampi, Anne Hyvärinen, Marina Erhola, Tari Haahtela, Kyösti Haukipuro, Ulla Haverinen-Shaughnessy, Kaisa Jalkanen, Kirsi Karvala, Sanna Lappalainen, Kari Reijula, Hannele Rämö, Markku Sainio, Anniina Salmela, Mika Salminen, Tuula Vasankari, Juha Pekkanen

**Affiliations:** 1 Finnish Institute for Health and Welfare, Helsinki and Kuopio, Finland; 2 Päijät-Häme Joint Authority for Health and Wellbeing, Lahti, Finland; 3 University of Helsinki, Helsinki, Finland; 4 Social Insurance Institution of Finland, Helsinki, Finland; 5 Tampere University, Tampere, Finland; 6 Finnish Institute of Occupational Health, Helsinki, Finland; 7 Finnish Housing Health Association, Helsinki, Finland; 8 Finnish Lung Health Association, Helsinki, Finland; 9 University of Turku, Turku, Finland

## Abstract

Clean and fresh indoor air supports health and well-being. However, indoor air can contain pollutants that can cause a variety of symptoms and reduce well-being. Individual exposure agents can also increase the risk of certain diseases. Finns have taken major steps to improve the quality of indoor air for several decades. The primary focus of these activities has been the prevention and reduction of exposure to poor indoor air quality through guidance and regulation directing remediation of damaged buildings. Nevertheless, reported symptoms related to poor indoor air quality are common in Finland. In addition to exposure to indoor air pollutants, this may be partly due to the lively public discussion on the health risks caused by poor indoor air quality, conflicting views between experts, and mistrust towards public authorities, building owners and builders. Because of the scale of the indoor air problems in Finland, people’s needs for reliable information and support, and the major costs involved, there is a call for new evidence-based methods, perspectives and solutions. Therefore, the Finnish Institute for Health and Welfare initiated the Finnish Indoor Air and Health Programme 2018–2028 together with a number of collaborators and stakeholders. The primary, long-term objective of the programme is to reduce hazards to health and well-being linked to indoor environments in Finland. To fulfill this objective, the programme will focus on the promotion of human health and well-being, the prevention of hazards, improved communication and engage the whole health-care sector to manage better patients´ symptoms and complaints. The 10-year Finnish Indoor Air and Health Programme consists of four areas that aim (1) to increase understanding of the effects of indoor environments on health and well-being; (2) to develop the management of problems linked to indoor environments; (3) to improve the treatment and working and functional capacity of people with symptoms and illnesses; and (4) to strengthen the competence in matters related to indoor environments. The progress of the programme and reaching the predefined, quantitative goals will be monitored throughout the programme.

## Background

Clean and fresh indoor air supports health and well-being. However, in some cases, indoor air can contain pollutants that may cause a variety of symptoms and reduce well-being. Individual exposure agents can also increase the risk of certain diseases [1–5]. According to the Finnish parliamentary Audit Committee, the annual cost of health impacts caused solely by exposure to indoor pollution linked to water damages is in the range of 23–953 million euros. In addition to this, there are large costs related to the repair of buildings [6].

Finns have taken major steps to improve the quality of indoor air for several decades. From the international standpoint, we are forerunners in both research and providing guidance on indoor air quality. The primary focus of these activities has been the prevention and reduction of exposure to poor indoor air quality through guidance and regulation directing remediation of damaged buildings. Water damage and visible mould are less common in Finland and also the levels of most other major indoor air pollutants are—on average—lower than levels found elsewhere in Europe, expect for radon [7–11]. However, in spite of this, reported symptoms related to poor indoor air quality are common in Finland [12]. In addition to exposure to indoor air pollutants, this may be partly due to the lively public discussion on the risk of indoor air exposures, conflicting views between experts and mistrust towards public authorities and building owners. Also environmental sensitivity to indoor air is prevalent [13, 14].

The issues related to indoor air are broad and complex, and improvements are required in several respects. Improvements are needed in the management of indoor air pollutants and in the prevention, management and communication related to problem situations. Those who suffer from symptoms in indoor environments or have illness are in need of help and support. The provision of diagnostic and treatment services, social security and other services that support rehabilitation still have shortcomings and must be improved. Improvements are also needed in communication, education and training.

Because of the large scale of the reported indoor air problems in Finland, people’s needs for information and support, and the large costs involved, there is a call for new evidence-based methods, perspectives and solutions. Therefore, in spring 2017, the Finnish Institute for Health and Welfare (THL) initiated the preparation of the Finnish Indoor Air and Health Programme 2018–2028 together with a number of collaborators and stakeholders, including the Finnish Institute of Occupational Health (FIOH). Programme was modelled based on the Finnish National Asthma Programme and National Allergy Programme that had already demonstrated successful cooperation in the management of these public health problems [15]. The content of the Finnish Indoor Air and Health Programme 2018–2028 is described in this report.

### Preparation of the programme

**Fig. 1:**
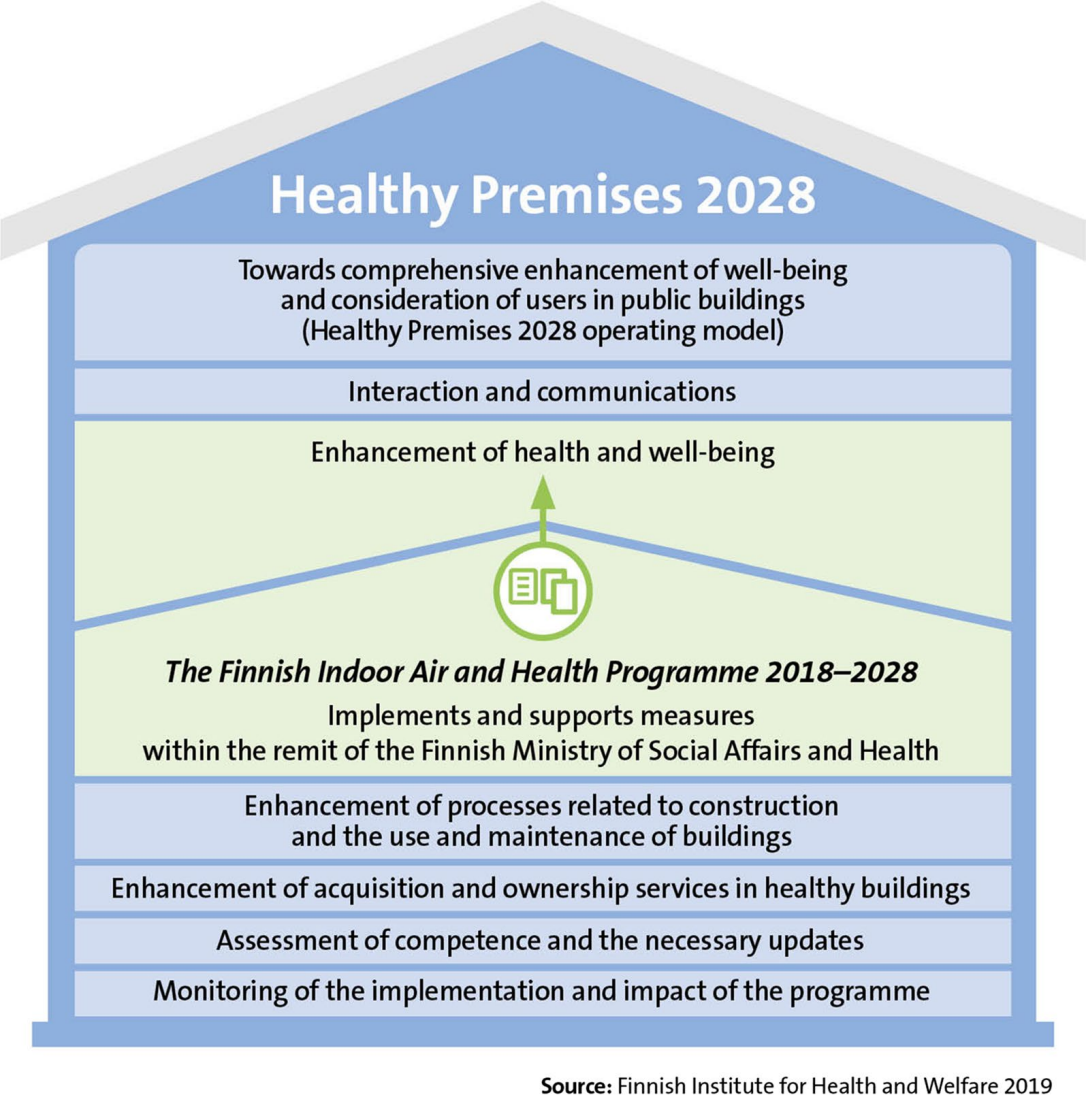
Main focus areas of Finnish Healthy Premises 2028 Programme. The Finnish Indoor Air and Health Programme implements and supports these measures (Enhancement of health and well-being) and closely collaborates with programme’s other actors

In autumn 2017, Prime Minister of Finland appointed a project group to draft the Finnish Healthy Premises 2028 Programme, aimed at making indoor air in public buildings healthy and enhancing care of all those with symptoms and illnesses resulting from poor indoor air quality. The drafting of the programme was preceded by a parliamentary round table discussion in June 2016, led by the Prime Minister. The Government approved the principle decision on the Finnish Healthy Premises 2028 Programme on 3 May 2018. Over the 10-year programme period, the Government’s extensive Finnish Healthy Premises 2028 Programme will function in seven different areas (Fig. 1). Responsibility for implementing the programme is divided between different Finnish ministries and administrative sectors and levels.

In spring 2017, the Finnish Institute for Health and Welfare (THL) initiated the preparation of the Finnish Indoor Air and Health Programme together with numerous cooperating bodies, including the Finnish Institute of Occupational Health (FIOH). The programme’s intention is to support the Finnish Government and the Finnish Ministry of Social Affairs and Health in the work to overcome adverse health effects related to poor IAQ. The Finnish Indoor Air and Health Programme, coordinated by the Finnish Institute for Health and Welfare (THL), will implement the measures to promote health and well-being listed in Chapter 3 of the Government’s Healthy Premises 2028 Programme that are the responsibility of the Finnish Ministry of Social Affairs and Health, and cooperate with other parties responsible for other areas in the programme.

The Finnish Indoor Air and Health Programme was designed by using the logical framework approach method, in collaboration with various stakeholders. Workshops during the preparation phase, work meetings, online surveys, and bilateral consultation with stakeholders were utilized in the planning process of the programme. A total of eight workshops and other major planning meetings were arranged in autumn 2017, during which a preliminary problem and objective analysis were carried out and a work plan was drawn up for the programme.

**Fig. 2:**
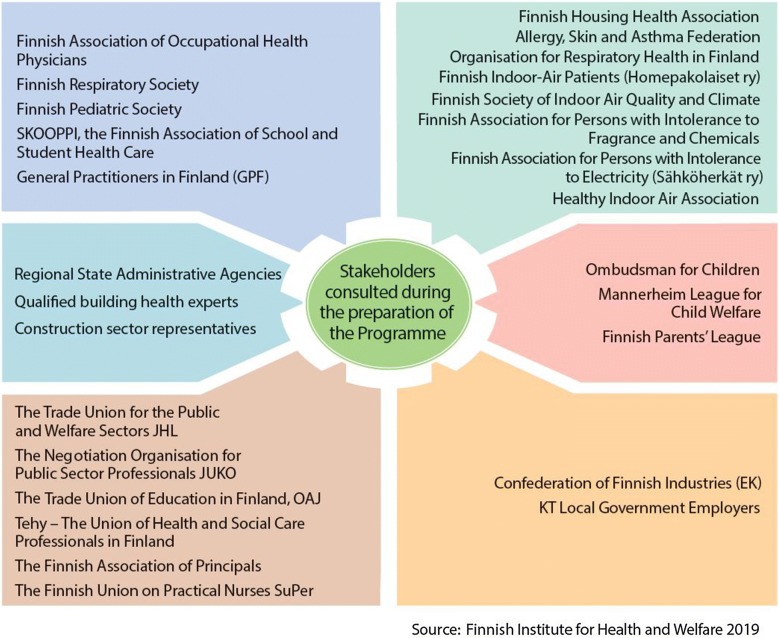
Stakeholders consulted during the preparation of the programme

During the design process, a knowledge base was compiled from evidence-based information, and two new reviews were conducted: (1) indoor air symptoms that limit people’s functional capacity and (2) the prevalence of indoor environmental exposures in Finland compared to the other Nordic countries. Furthermore, indoor air experts from Sweden, Norway, Denmark, and Iceland were interviewed. Key stakeholders were also widely consulted during the drafting of the programme (Fig. 2). Topics raised during these consultations, such as the prioritization and shortcomings of measures and the willingness of different parties to participate in implementing the programme were used during its planning stage. At the end of the preparation, in spring 2018, a work meeting was held for key experts and stakeholders. The design process led to the final structure of programme and defined both the objectives for 2018–2028 and the measures needed for achieving them. The draft programme was published in September 2018, and a comment round for key stakeholders on the content of the draft, as well as, a survey open to public were organized. The survey results were used in finalizing the programme and will be utilized in its implementation. The programme will further take shape during its implementation via intensive collaboration with the key actors involved.

### Key problems with the current state

**Fig. 3:**
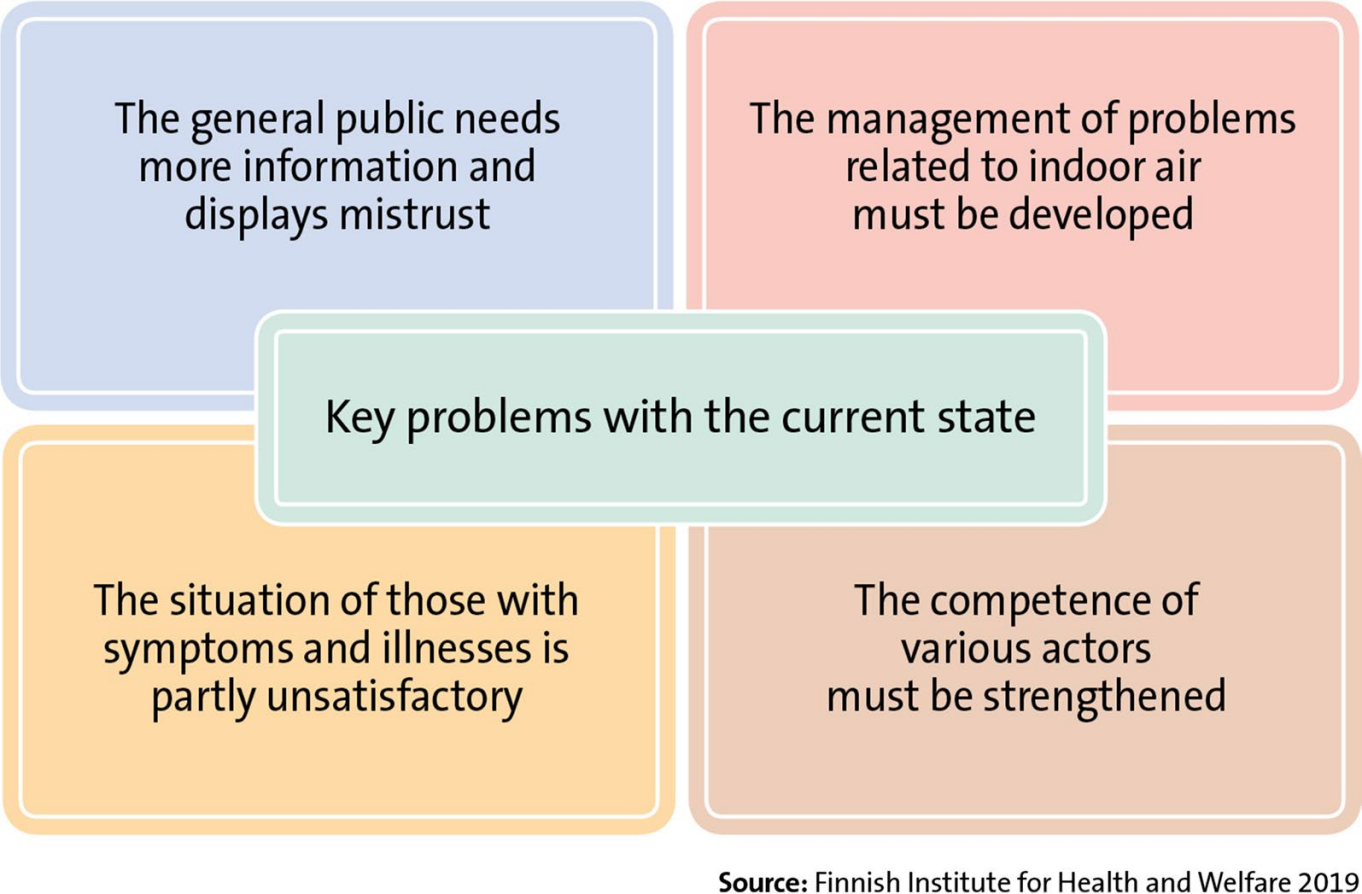
Key problems with the current state and the aspects on which the Finnish Indoor Air and Health Programme will focus. The situation also requires measures on enhancement of processes related to construction, use, and maintenance of buildings

The problems related to indoor air quality are broad and complex and development is required in numerous areas, such as in the construction and maintenance of buildings, processes related to handling indoor air quality problems in buildings, care and support provided to people, and education and training (Fig. 3). These problems were identified at workshops during the preparation phase of the Finnish Indoor Air and Health Programme, at stakeholder meetings, from online surveys, during bilateral stakeholder consultations, and by interviewing Nordic specialists. Resolving the issues and problems listed above also calls for more research.

People in Finland are less exposed to moisture damage in buildings and to other most common impurities in indoor air than other Europeans on average, expect for radon. The situations vary, however, and there still are indoor air quality related problems that must be dealt with; much work remains to be done in Finland to improve indoor air quality and indoor environments. Measures supporting the development of processes related to construction, use and maintenance of buildings, are primarily implemented by parties in the Finnish Healthy Premises 2028 Programme other than actors of the Finnish Ministry of Social Affairs and Health. However, these measures must be executed by paying attention to the promotion of health and well-being.

Numerous instructions and guidelines have been prepared for resolving problems related to indoor environments in buildings. However, there is still plenty of room for improvement in the management and adoption of the guidelines and instructions. Not all municipalities have coherent and clear operating instructions for handling issues related to indoor air quality. There may be ambiguities in the division of tasks and not enough attention has been paid to communications. The resolution of indoor air quality issues should be based on a systematic, well-organized and multiprofessional process, with a clear specification for the division of tasks and responsibilities. Furthermore, legislation on housing health and workplaces is not fully consistent, so measures related to indoor air quality issues are contradictory at times (such as decisions concerning students and teachers at schools) [16]. Furthermore, cooperation between public authorities and different administrative sectors should be increased.

The assessment of the exposure conditions in indoor environments and characterization of the related health effects should be further developed and re-evaluated. Some degree of moisture damage and impurities are commonplace in all types of buildings, but assessing the health-related significance of moisture damages is extremely complicated. For instance, there are no health based limits for microbial exposure that could be used for evaluating building-related measures and their urgency. The interpretation and use of indoor air questionnaires for school children remain unestablished. Improving the exposure assessment and health risk evaluation enables rational management of problem situations and the building stock so that the resources available can be allocated most effectively in terms of reducing health hazards and prevention, while increasing the trust of users and the general public in the management of problem situations and the safety of indoor environments.

The situation of patients with symptoms and illnesses is partly unsatisfactory. There may be shortcomings and development needs in care and service paths, diagnostics and treatment, operating models that support people with symptoms, social security, and for service packages that support rehabilitation. Furthermore, people feel that health care professionals have inadequate knowledge and skills for working with patients with symptoms linked to indoor environments. The care of patients suffering from environmental sensitivity must be studied and developed because there are no efficient practices for treatment and rehabilitation.

The health effects of indoor air quality are of great concern, and related complaints and symptoms are common. Such situations are sometimes exacerbated by significant uncertainty and mistrust towards public authorities and building owners. There is a lively public discussion on indoor air quality issues, which is occasionally escalated and portrayed in very black-and-white terms, with various specialists presenting different views of the health hazards of impurities in indoor air. This can be reflected in the general public’s knowledge and attitudes, and it should be quickly analyzed. Such reports should be used as guidelines for improving the situation, starting from improved supply of information and increased open communication that generates trust.

Solving problems related to indoor air quality and supporting people with symptoms requires new methods and solutions based on evidence-based information, highlighting human health and well-being. All factors impacting the situation should be reviewed, not merely impurities in indoor air.

### Objectives and activities

**Fig. 4:**
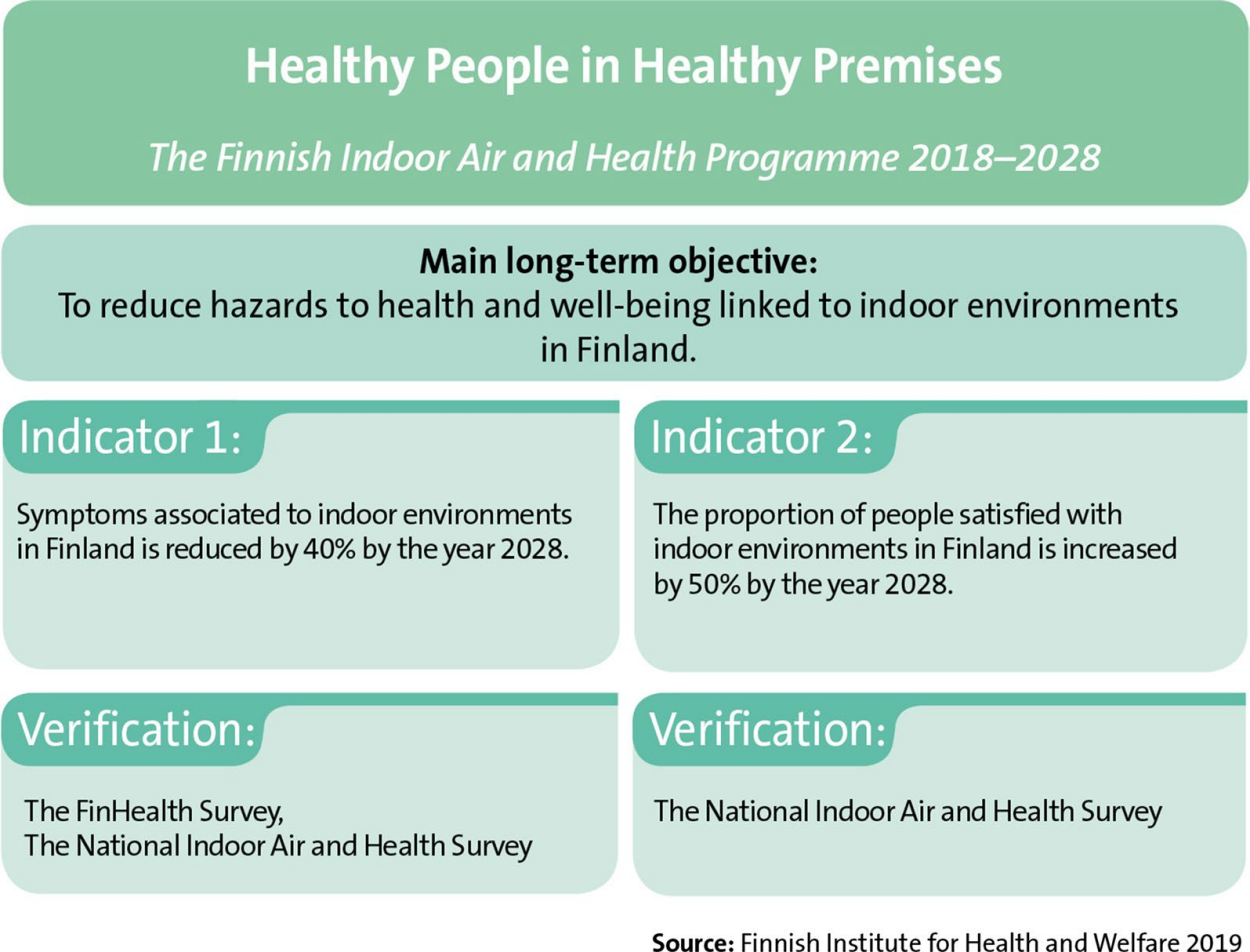
The primary long-term objective of the Finnish Indoor Air and Health Programme

**Fig. 5:**
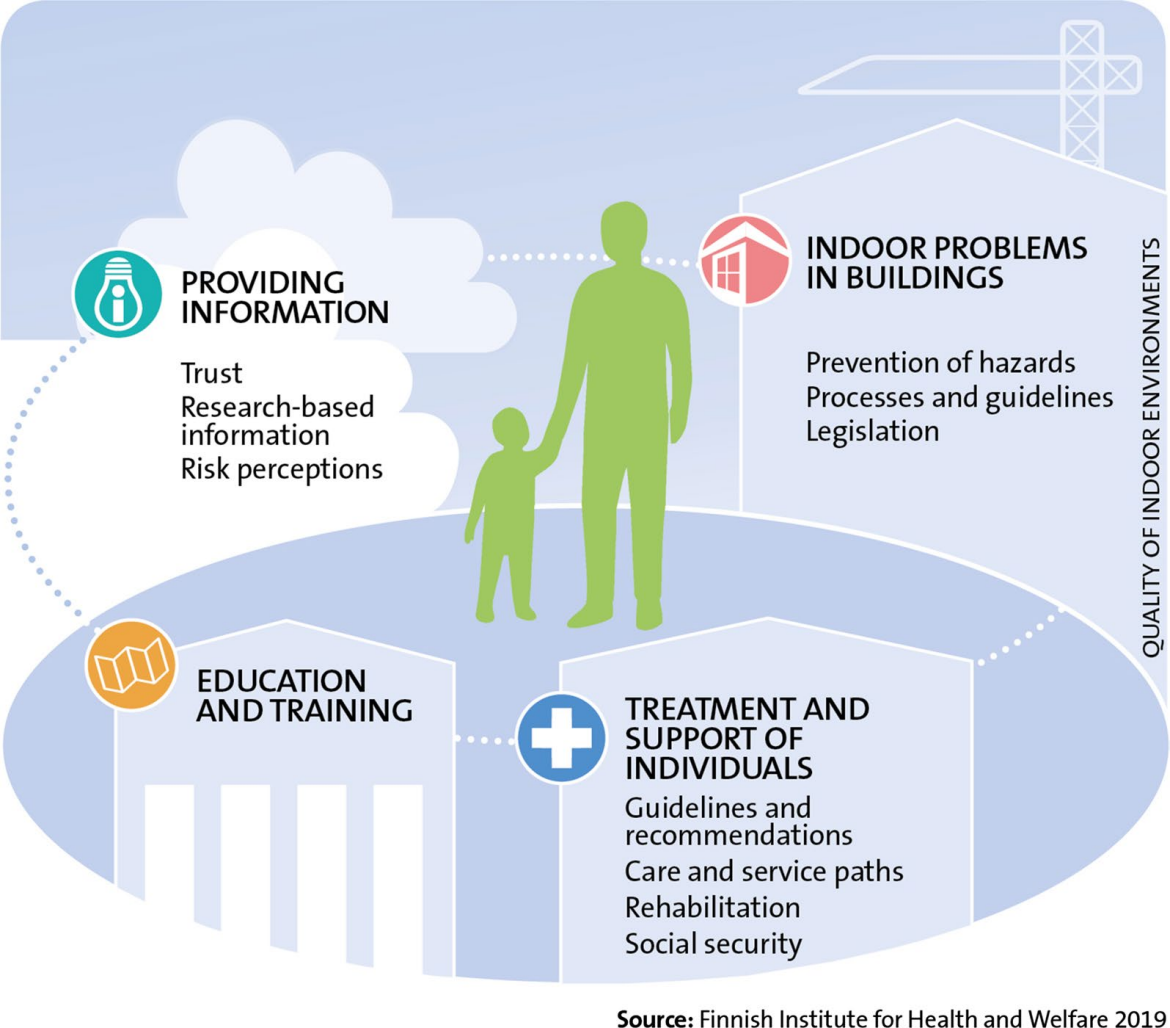
The Finnish Indoor Air and Health Programme develops new evidence-based methods, perspectives and solutions for solving problems related to indoor air and for treating and supporting people with symptoms and illnesses. The programme will focus on promotion of human health and well-being, prevention of hazards, communication, and comprehensive treatment and support of people who are ill

The primary long-term objective of the Finnish Indoor Air and Health Programme is to reduce hazards to health and well-being linked to indoor environments in Finland (Fig. 4). The method for achieving this is to highlight human health and well-being (Fig. 5). The purpose of the programme is to develop the management of problems linked to indoor environments, improve the working and functional capacity of people with symptoms and illnesses and to increase understanding of the effects of indoor environments on health and well-being. Attaining the programme’s long-term objective requires also actions related to the enhancement of construction processes and building maintenance. Furthermore, research should support the achievement of programme objectives.

The Finnish Indoor Air and Health Programme consist of four areas: providing information, indoor problems in buildings, treatment and support of individuals and education and training (Fig. 5). The actions in these areas aim to respond to the key problems with the current state (Fig. 3). The contents of these areas are described, in detail, in the following sections. Many actions of the Finnish Indoor Air and Health Programme do not make a distinction between public and other buildings. How the programme’s objectives are achieved is monitored by indicators created for them. These indicators are compiled from surveys directed at the general public and municipal actors that are repeated at least at the beginning and end of the programme. In addition to these indicators, outcome indicators are used to describe how the planned actions are realised.

### Area 1: Providing information

**Fig. 6:**
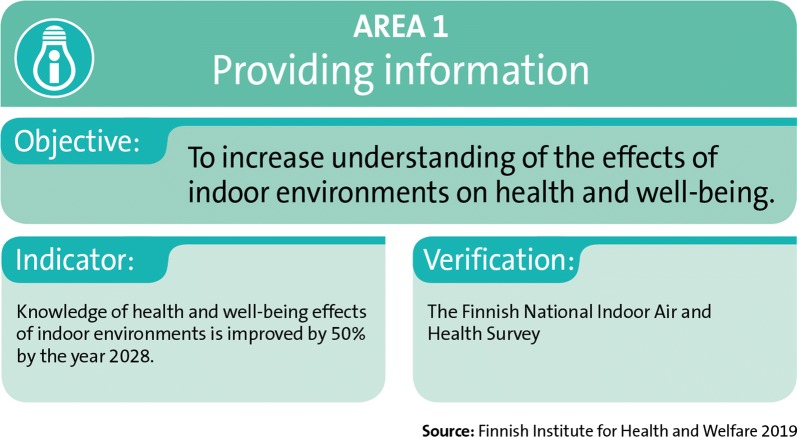
The objectives, indicators, and verification sources for area 1

The objective of the actions in area 1 is to increase the general public’s understanding of the effects of indoor environments on health and well-being (Fig. 6). The activities of area 1 are described in the sections below. Influencing public understanding by providing information and communications are also key elements in all the other areas of the Finnish Indoor Air and Health Programme. Area 2 focuses on communications in problem situations with buildings, area 3 distributes information for people with symptoms and illnesses and area 4 provides training on communication skills.

#### Assessing the public’s knowledge and attitudes on health risks related to indoor air quality in Finland

In autumn 2018, a comprehensive public survey was conducted on the public’s knowledge and attitudes related to indoor air, and their perceptions of health hazards, and levels of trust. The information obtained was used for identifying target groups and communication topics in cooperation with professionals from different fields of science. The results are used for considering how to improve risk perceptions and to fill in any gaps in the information. The results are drawn up in a report, and the observations are communicated actively.

#### Reviewing and updating communication materials and communicating actively

Communication actions will ensure that the perceptions of the general public and various actors of the effects of indoor environments on health and well-being match the scientifically derived information. The work will be started by drawing up a communication plan in cooperation with the Finnish Healthy Premises 2028 Programme. The timeline of the communication plan is monitored regularly and updated whenever necessary. Parties communicating on indoor air issues are mapped, and a “network of communicators” is launched (e.g., associations, organizations in the health sector). Furthermore, target groups for the initial phase of health communications are being analyzed, and messages based on researched information in line with the key contents of the programme are developed for the identified target groups.

Material packages are prepared for online use by the various target groups; the subjects include for example indoor air and health and information about indoor air for workplaces. Organizations in the social welfare and health care sector will use material packages based on researched information to tailor information which is in line with the programme’s core messages for their own target groups. In addition, the messages will be actively communicated by using all appropriate channels; new communication materials are being prepared; and a comprehensive, national communication programme on the health impacts of indoor air, based on research evidence, will be designed. These actions will also improve the general public’s ability to find appropriate information and the correct party to contact in case of any problems.

### Area 2: Indoor problems in buildings

**Fig. 7:**
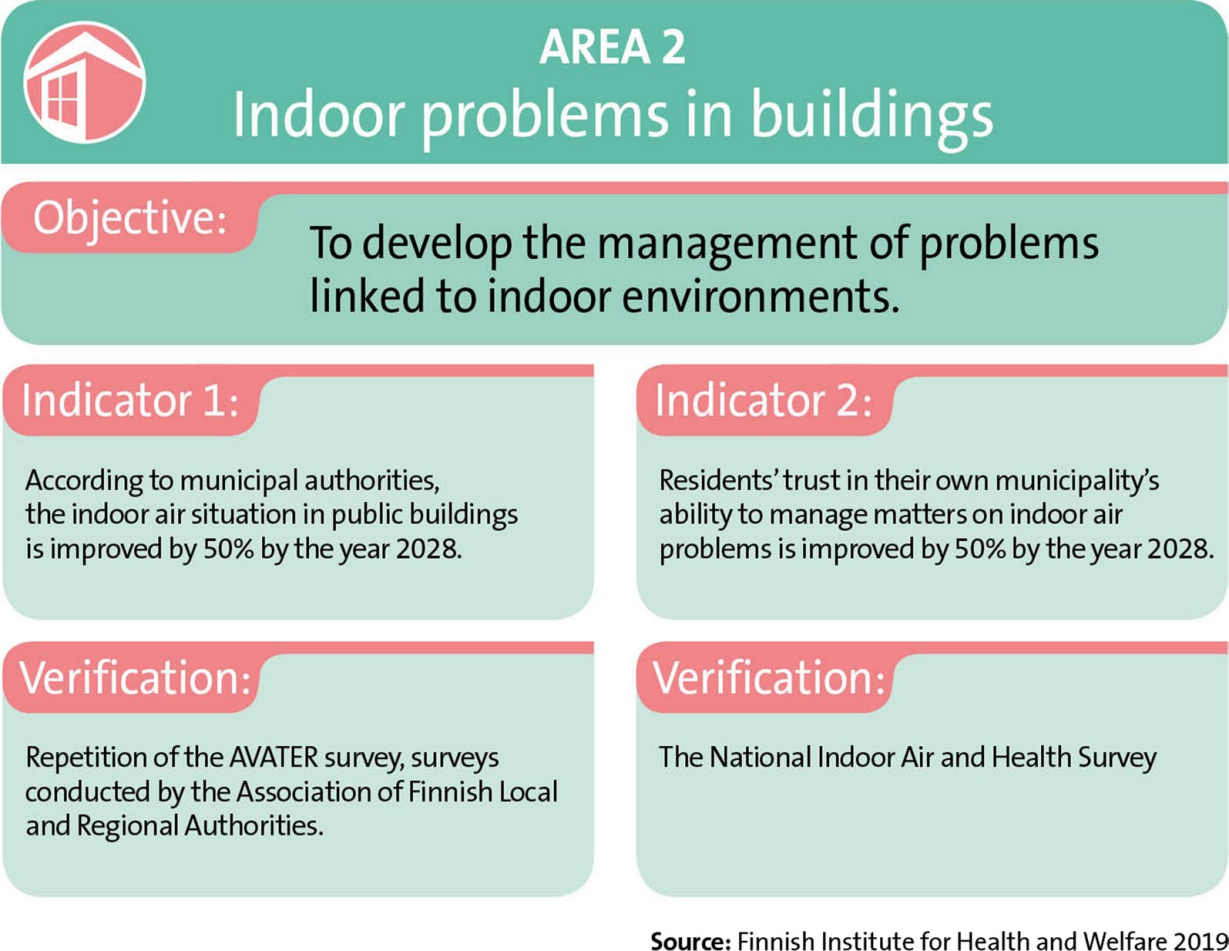
The objectives, indicators, and verification sources for area 2

The objective of the activities associated with area 2 is to develop the management of problems linked to indoor environments (Fig. 7). The actions included in area 2 are described in the sections below. The actions described are performed in close cooperation with the Finnish Healthy Premises 2028 Programme and, if necessary, in discussions with the ministries responsible for them.

#### Developing processes for management of indoor air problem situations with buildings

Instructions describing the management of situations with buildings are being brought together and further improved. New instructions will be drawn up if needed. The work will be started by assessing the existing information. Initially, this means reviewing the various guidelines, roles and duties related to resolving problem situations, analyzing possibilities for cooperation, and searching for good practices that are currently in use. Furthermore, process descriptions on managing different levels of problem situations in buildings are developed. Management processes for indoor air problems in schools, day care centers and private homes are prepared or supplemented. These initially focus especially on rapid intervention in problem situations. Resolution processes are developed by analyzing the best models of electronic systems associated with process management and ideas for their further development. In addition to this, methods are determined for improving open communications, consultations with users of buildings, guided participation in the management of indoor air matters, and the production of information concerning buildings.

Indoor air quality recommendations are compiled to summarize the guidelines based on researched information and good practices. Additionally, new instructions are needed for example on the use of temporary facilities, cleaning of homes after moisture and mould damage, and decontamination of movable property. The production of information is partially parallel to the public communication in area 1, but the target groups in area 2 are actors specifically related to the management of problem situations in buildings.

#### Developing the assessment of exposure conditions

Indoor air quality is affected by numerous factors, such as outdoor air quality, building and ventilation technology, activities on the premises, physical factors, and chemical and biological factors. Assessment of exposure conditions must be based on a comprehensive building investigation, reviewing the effects of building characteristics and effects of its structure and systems. In the development of an assessment model on exposure conditions by the Finnish Institute of Occupational Health (FIOH), consistent and coherent operating models are being created for evaluating exposure conditions at workplaces, in homes, schools and day care centers, and their adoption will be ensured. A more comprehensive assessment of exposure conditions requires detailed guidelines on studying and interpreting microbes, particles and chemical compounds. In addition, methods for measuring impurities in indoor air, national evaluation practices, definitions, concepts and instructions must be further developed, and details on approved methods must be brought together.

#### Develop assessment of health risk evaluation

The assessment of health risk evaluates the risk to health caused by a building’s exposure conditions. It involves assessment of exposure conditions, evaluating the likelihood of exposure, and the health effects of exposure agents. Other areas reviewed include users’ symptoms and illnesses, exposure times, and avoiding harmful exposure.

The Finnish Indoor Air and Health Programme develops operating models and guidelines for assessing the significance of indoor air quality problems in buildings at workplaces, homes, schools and day care centers, and supports their systematic adoption. The development efforts take account of the different user groups, such as children and working-aged people. The current operating models, guidelines, information and methods need to be further developed and harmonized, and the experts implementing them need to be trained. Indoor air quality questionnaires are developed and their role in the assessment of health risk evaluation is clarified. The programme will also study possibilities for providing health protection authorities with medical support in analyzing and solving problem situations.

#### Reducing contradictions in legislation and making help more available to the general public

As the processes concerning problem situations in buildings are developed, it is necessary also to study and assess the timeliness and possible shortcomings and contradictions of current legislation. It is also necessary to analyze practical differences in interpretation, such as in procedures linked to health protection legislation as well as to occupational safety and health legislation concerning matters related to indoor air [16]. The Finnish Indoor Air and Health Programme is taking part in the analysis of the Finnish Healthy Premises 2028 Programme on the timeliness, shortcomings, mutual contradictions and practical interpretation differences in legislation. Proposals for changes will be submitted on the basis of this work, if necessary. To make help more accessible for the general public, the programme will analyze the options for obtaining state subsidies for repair costs caused by problems with indoor air quality in buildings.

### Area 3: Treatment and support of individuals

**Fig. 8:**
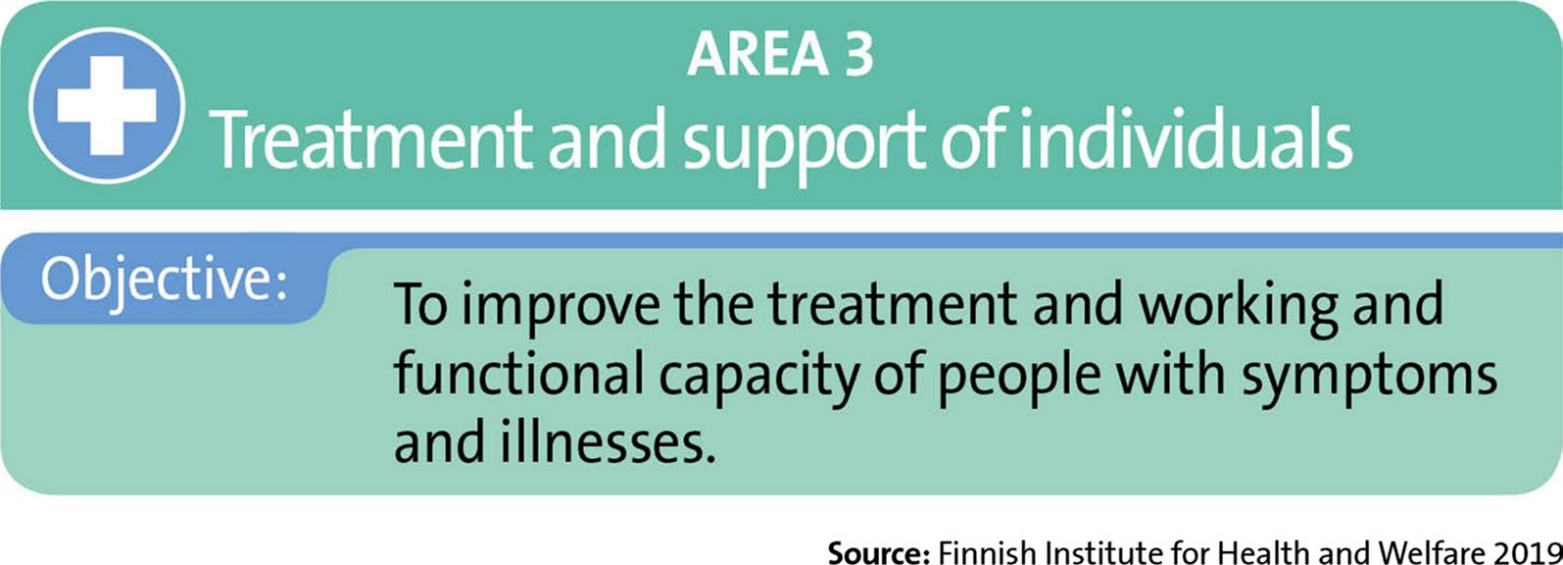
The objective of area 3

Actions linked to the treatment and support is aimed at improving the treatment and working and functional capacity of people with symptoms and illnesses (Fig. 8). These actions are directed at the entire, broad variety of health risks associated with indoor air.

The actions included in area 3 are described in the sections below. The objective of area 3 is measured using outcome indicators, i.e., the realization of the actions designed for it. The production of information and communication in area 3 are partially parallel to the public communications in area 1, but the target groups in area 3 are specifically patients and health care professionals.

#### Developing prevention, treatment and rehabilitation at all levels of care and service

Activities linked to this objective are intended to describe the range of services in social welfare and health care available to those with symptoms in indoor environments and to develop those services. Furthermore, under this objective, a national development plan for treatment and support will be created, in order to support treatment and rehabilitation at all care and support levels. For instance, the health care system and care paths must support the health and rehabilitation of people with symptoms associated with indoor environments. Care and service paths are being reviewed for different target groups, such as children and working-age people. Health care will also be better informed on the differential diagnosis of indoor air-associated symptoms and illnesses. At the same time, it is being determined if primary health care needs support treating people with symptoms in indoor environments and whether this can be arranged (e.g. possibilities for consultation).

#### Bringing together information and guidelines that support health and well-being

More information that supports both health and well-being is needed for all target groups and at all levels of operation. For this purpose, the programme will bring together information for people with symptoms and illnesses and for those who have experienced symptoms associated with indoor environments. This information will be published, to be accessed by everyone. Also information materials and tools for health care professionals will be published. Support and guidance provided to people suffering due to the poor indoor quality air as well as those who suspect indoor air quality issues and help for them will be improved to convey accurate information and good practices.

#### Developing treatment and rehabilitation methods and support for patients with severe symptoms associated with indoor environments

Some people with severe symptoms and illnesses need support that is more comprehensive than provided by primary health care in order to improve their working and functional capacity. Such broader support could be provided by clinics that treat and rehabilitate patients in cooperation between various health care sectors. Rehabilitation may also require individual solutions. The Finnish Indoor Air and Health Programme supports the establishment of rehabilitation outpatient clinics for patients who have symptoms associated with indoor environments and who’s working and functional capacity has been severely impaired. The support will involve the development of treatment and rehabilitation methods for treating environmental intolerance and preparation of evidence-based clinical practice guidelines for the treatment and rehabilitation of functional symptoms and disorders linked to indoor environments. Additionally, proposals will be prepared for developing multidisciplinary rehabilitation and access to rehabilitation. The grounds for diagnostic procedures on occupational diseases related to exposure agents in indoor air will be analyzed and assessed.

#### Assessing the development needs of income security to support the rehabilitation of people with symptoms linked to indoor environments

The work linked to this objective involves participating in the preparation of work by the Finnish Healthy Premises 2028 Programme on the social security of patients with symptoms linked to indoor environments. Its objective is to analyze the development of income security that would support the rehabilitation of people with symptoms linked indoor environments and their equal treatment with other client groups.

### Area 4: Education and training

**Fig. 9:**
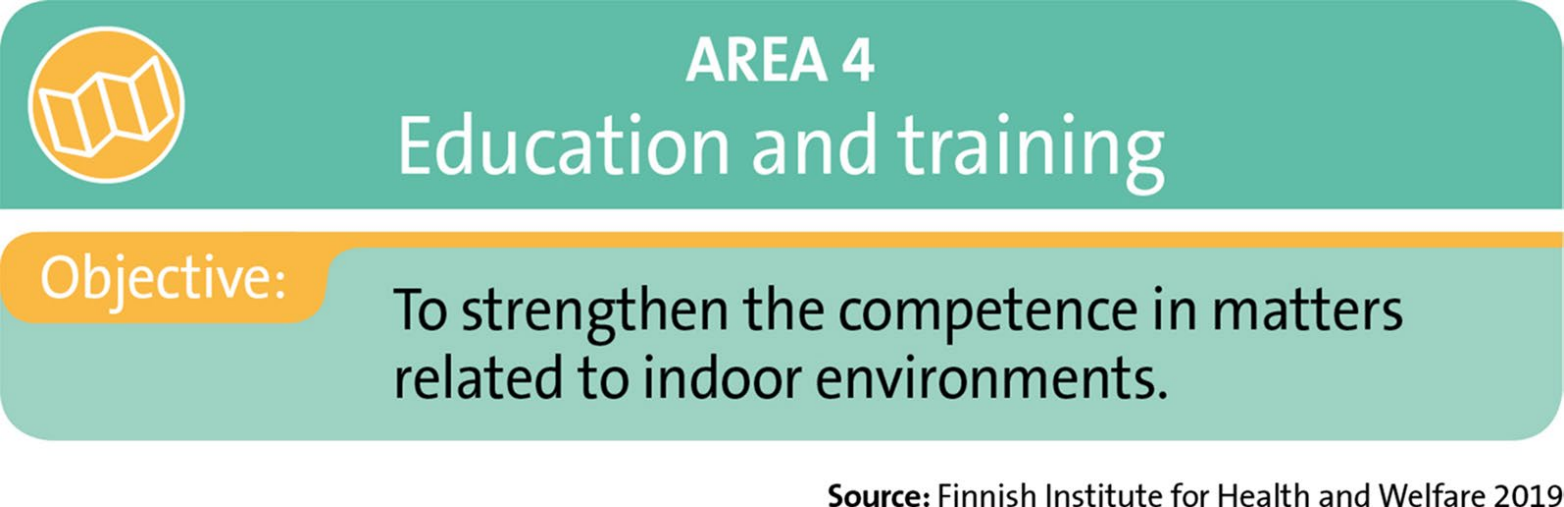
The objective of area 4

The objective of the activities associated with area 4 is to strengthen the competence in matters related to indoor environments (Fig. 9). The actions of this area are described in the sections below. The objective of area 4 is measured with outcome indicators, i.e., the realization of the actions designed for it.

#### Increasing education and training at all different levels of operation

Increasing training on health risks linked to indoor environments, as well as, processes and best practices, improves and enhances the competence of the various parties. This work is done in collaboration with the Finnish Healthy Premises 2028 Programme and involves surveying the need for education and further training of different actors (including property owners, building managers, and property maintenance staff). In addition, a report is compiled, focusing on the need for training to social welfare and health care professionals. Principals and managers of the day care centers will be provided with training on how to manage processes involving indoor air quality problems. Training on good practices will also be given to health protection and occupational safety and health authorities, indoor air working groups, and other actors identified in surveys. The Finnish Institute of Occupational Health (FIOH) provides and develops training on the indoor environment at workplaces for indoor climate experts, occupational health professionals, and employees and parties who support them.

#### Actively provide training in communication skills

Various parties’ skills in communicating problem situations concerning indoor environments will be developed by means of instructions and training events. Communication will be developed by clarifying the terminology related to indoor air. Furthermore, an operating model is developed in order to help actors at workplaces as well as health protection and occupational health and safety authorities streamline their cooperation and communicate their decisions together.

### Assumptions, constraints and risks

The achievement of the objectives of the Finnish Indoor Air and Health Programme, and the realization of its measures involve significant risks. For the successful realization of the programme, it is necessary to obtain sufficient funding and skilled experts for the various areas of the programme. It is also important that the different parties are committed to the cooperation, and that measures in these areas receive the necessary political and other support.

The most significant assumptions in terms of the objectives involve the impact of the measures. The objectives of the Finnish Indoor Air and Health Programme can be attained if all the areas of the project are implemented in cooperation, and if the measures really have the impacts outlined in the programme’s objectives. Different stakeholders must share a common view of the problems and measures needed for solving them and must work together to make that view a reality. However, even this does not guarantee that the quantitative objectives set for the programme are achieved as it is difficult to assess the impact of the various actions. Changes in quantitative objectives can be strongly influenced by many factors outside the programme.

**Fig. 10:**
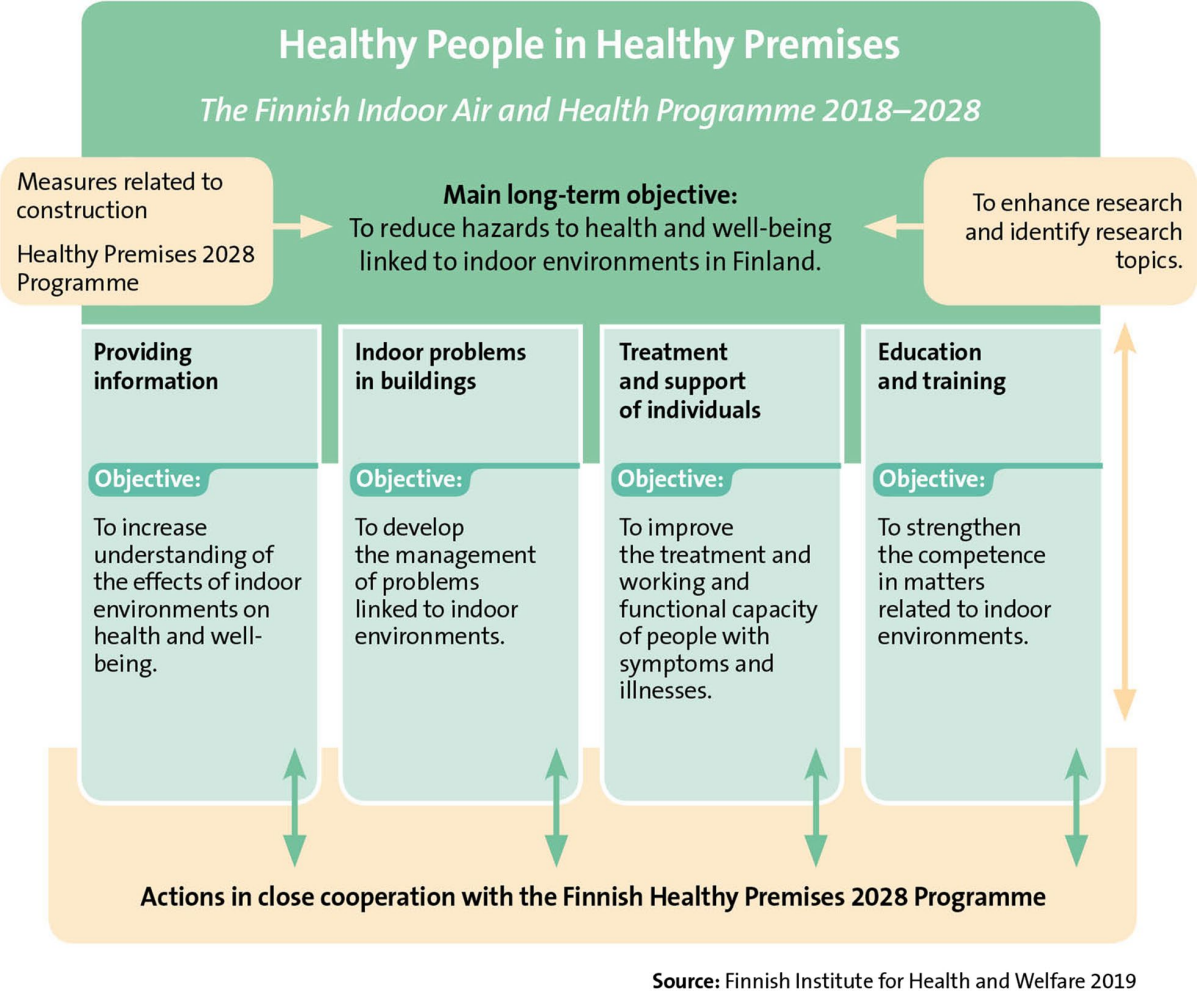
The attainment of the programme’s primary objective is also affected by measures related to construction, which are implemented by administrative sectors other than the Finnish Ministry of Social Affairs and Health taking part in the Finnish Healthy Premises 2028 Programme. The enhancement of research would also support the achievement of the programme objectives

The attainment of the programme’s primary objective will also be affected by measures on the enhancement of processes related to the construction, maintenance, operation and use of buildings, which are implemented by administrative sectors other than the Finnish Ministry of Social Affairs and Health taking part in the Finnish Healthy Premises 2028 Programme (Fig. 10). This means that the operation and continuation of the Finnish Healthy Premises 2028 Programme holds an important position in the achievement of the objectives of the Finnish Indoor Air and Health Programme. Furthermore, although conducting research studies on the indoor air is not directly an objective of the programme (Fig. 10), significant enhancement of the research would support the measures and thus help to achieve the quantitative objectives of the programme.

### Stakeholders and beneficiaries

**Fig. 11:**
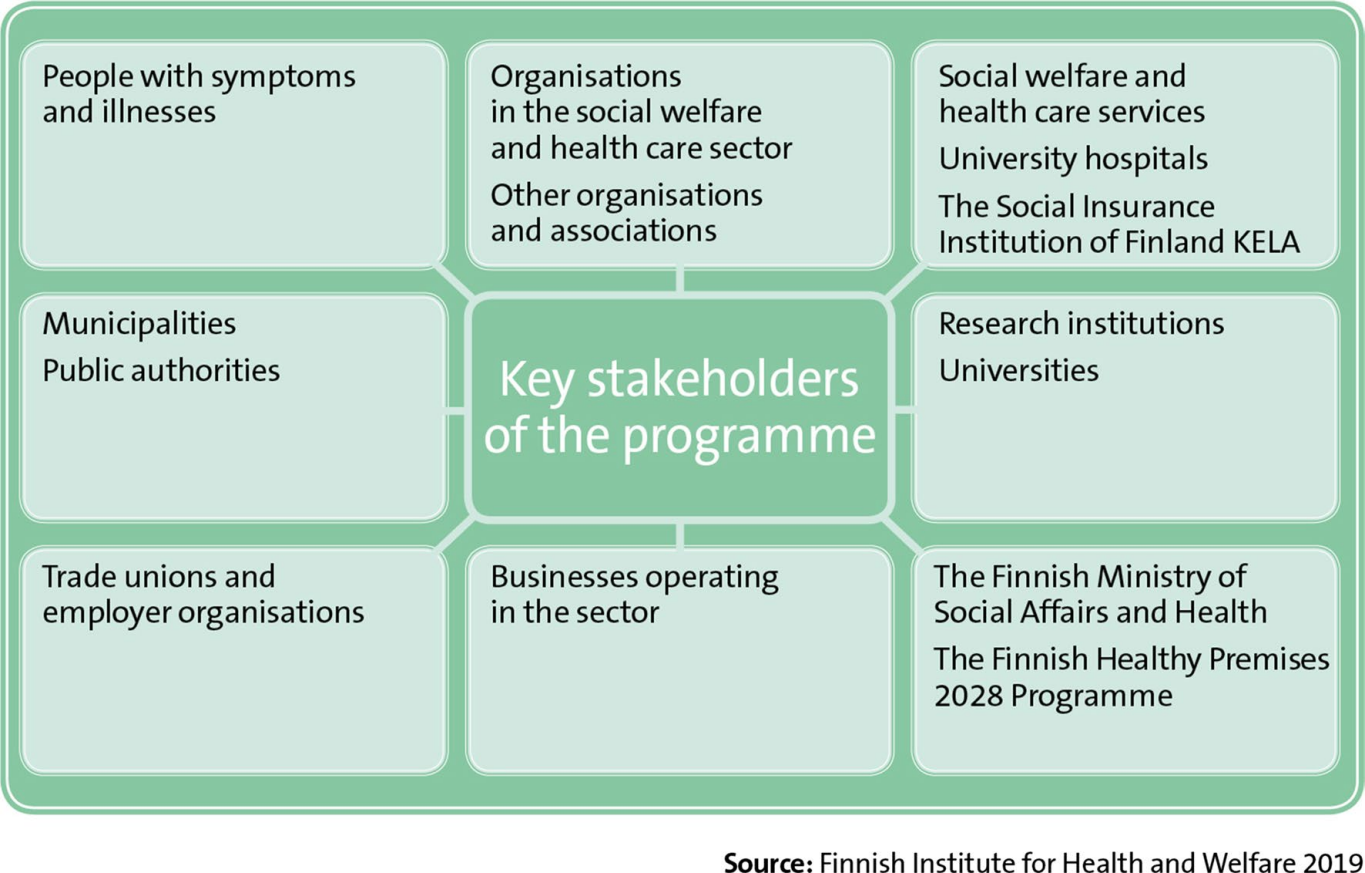
Key stakeholders of the Finnish Indoor Air and Health Programme

**Fig. 12:**
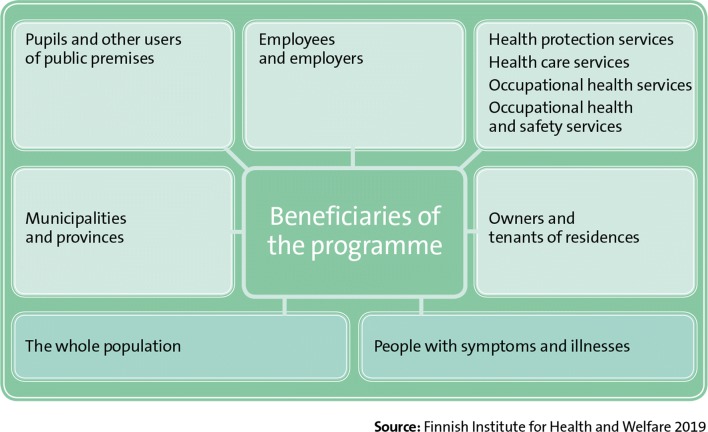
Beneficiaries of the Finnish Indoor Air and Health Programme

The programme’s key stakeholders (Fig. 11) are people who suffer from symptoms and illnesses, organizations in the social welfare and health care sector, social welfare and health care services, occupational health and safety professionals, health protection services, municipalities, other authorities, research institutions, businesses, the Social Insurance Institution of Finland, trade unions and employer organizations, the Finnish Ministry of Social Affairs and Health, and the Finnish Healthy Premises 2028 Programme, among others. Beneficiaries of the programme (Fig. 12) include all people who have symptoms associated with indoor environments, including school pupils and other users of public premises, employees and employers, municipalities and provinces, health care services, occupational health care services, health protection services, occupational safety and health services, and ultimately the whole population.

Social welfare and health care services provide treatment and rehabilitation for people with symptoms and illnesses. Organizations in the social welfare and health care sector promote the interests of patients who have symptoms or illnesses associated with indoor environments, and also provide guidance and advice to people with symptoms. Research institutions and universities produce and share information and guidance based on research in indoor air, develop processes, and provide expert support. Businesses use and apply the information and guidelines produced in solving indoor air quality problems. The Social Insurance Institution of Finland’s mission is to manage the social security of people living in Finland during different situations in life. The activities of public authorities include the supervision and steering of compliance with indoor air guidelines. Trade union and employer organizations supervise the benefits of employees in their respective sectors. The duty of the Finnish Ministry of Social Affairs and Health is to promote the good health and functional capacity of the public, to help create a healthy working and living environment, as well as secure sufficient social welfare and health care services. The objectives of the Finnish Healthy Premises 2028 Programme are to make public buildings healthy and to enhance the care and rehabilitation of all those who suffer from symptoms associated with indoor environments.

### Phasing of programme measures and monitoring its objectives

**Fig. 13:**
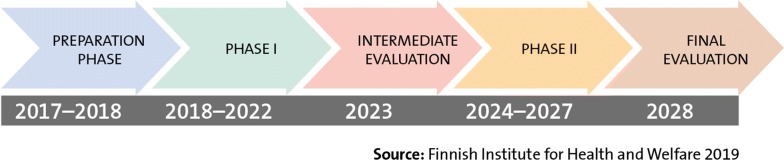
Design and evaluation of the programme, and phasing of its measures

According to the current plan, the measures of the Finnish Indoor Air and Health Programme will be implemented in two phases (Fig. 13). Measures in phase I will be launched in 2018–2022. Phase I includes the launch of measures that were deemed to be the most important or most impactful during the design phase.

The plan calls for an evaluation of the progress of programme measures and the implementation of its objectives in an intermediate evaluation in 2023. At that point, it will be determined how many of the planned measures have been realized, and it will be assessed as to how well the objectives set for the programme are proceeding. The progress of the measures and objectives will be documented and reported in the intermediate evaluation.

The measures for phase II will be designed and scheduled in detail in the intermediate evaluation (in 2024–2028). At the same time, it will be considered whether new methods or measures are required for implementing the objectives of the programme. In the end of the programme, a final evaluation will be prepared to determine how well the objectives and measures created for the programme have been realized, and proposals for further action will be submitted.
